# Macrophage Foam Cell-Targeting Immunization Attenuates Atherosclerosis

**DOI:** 10.3389/fimmu.2018.03127

**Published:** 2019-01-10

**Authors:** Fazhan Wang, Zhi Zhang, Aiping Fang, Quansheng Jin, Dailong Fang, Yongmei Liu, Jinhui Wu, Xiaoyue Tan, Yuquan Wei, Chunling Jiang, Xiangrong Song

**Affiliations:** ^1^State Key Laboratory of Biotherapy, Department of Anesthesiology and Translational Neuroscience Center, West China Hospital, Sichuan University and Collaborative Innovation Center for Biotherapy, Sichuan University, Chengdu, China; ^2^School of Chemical and Pharmaceutical Engineering, Sichuan University of Science and Engineering, Zigong, China; ^3^West China School of Public Health, Sichuan University, Chengdu, China; ^4^Department of Pathology/Collaborative Innovation Center of Biotherapy, Medical School of Nankai University, Tianjin, China

**Keywords:** atherosclerosis, macrophage foam cells, immunization, whole-cell vaccine, humoral immune responses

## Abstract

**Background:** Macrophage foam cells (FCs) play a crucial role in the initiation and progression of atherosclerosis. Reducing the formation or inducing the removal of FCs could ameliorate atherosclerosis. The present study examined whether the whole-cell vaccination using FCs could be used as novel prevention and treatment strategies to battle atherosclerosis.

**Methods:** ApoE^−/−^ mice with initial or established atherosclerosis were subcutaneously immunized three times with FCs in Freund's adjuvant.

**Results:** Immunization with FCs resulted in an overt reduction of atherosclerotic lesion in the whole aorta and the aortic root with enhanced lesion stability. Subsequent study in mechanism showed that FCs vaccination dramatically increased CD4^+^ T cell and CD8^+^ T cell populations. Immunization with FCs significantly raised the plasma FCs-specific IgG antibodies. Of note, the FCs immune plasma could selectively recognize and bind to FC. FCs immune plasma significantly blocked the process of FCs formation, finally reduced the accumulation of FCs in plaque. Additionally, it was observed that FCs immunization down-regulated the expression level of atherosclerosis related pro-inflammatory cytokines, including IFN-γ, MCP-1, and IL-6 and enhanced the lesion stability with a significant increase in TGF-β1 level and collagen content.

**Conclusions:** These findings demonstrate that the whole-cell vaccination using FCs significantly decreased lesion development and positively modulated lesion progression and stability by targeting FCs. The whole-cell FCs vaccine might represent a potential novel strategy for development of new antibodies and vaccines to the prevention or treatment of atherosclerosis.

## Introduction

Atherosclerosis is the most common pathological cause leading to cardiovascular disease which accounts for 17.3 million global deaths per year (1). Despite an increasing urgency to conquer atherosclerosis, its treatments are currently limited to lipid-lowering therapy and anti-platelet therapy (2). No more than 30–40% of the major cardiovascular events could be prevented by these strategies, probably attributing to the atherosclerosis-associated complications. The complications were mainly caused by the rupture-prone vulnerable plaques (3). In this sense, alternative treatments for atherosclerosis such as immune therapy have come into play (4–6).

Atherosclerosis is a chronic inflammatory disease involving accumulated modified lipids (oxygenized low density lipoprotein, oxLDL), macrophages, FCs, dendritic cells, inflamed smooth muscle cells, and endothelial cells (7–9). In the early stages of atherosclerosis, oxLDL accumulates in the artery intima. The oxLDL then induces dysfunction of endothelial cells and smooth muscle cells, which causes the production of pro-inflammatory cytokines (10). The circulating monocytes adhere to activated endothelial cells and subsequently migrate into the subendothelial space in response to locally produced chemoattractant molecules. These monocytes further differentiate into macrophages which could take up oxLDL via scavenger receptors (11, 12). The pathogenic macrophages transform into FCs and form the fatty streak, and then produce a diverse repertoire of inflammatory mediators that exacerbate disease (10). Accumulation of excessive oxLDL-derived cholesterol induces FCs apoptosis, thereby leading to the formation of a necrotic, cholesterol-rich core. The necrotic core becomes walled off by a fibrous cap of extracellular matrix proteins secreted by smooth muscle cells (11).

Reducing the formation of FCs or inducing the removal of FCs could ameliorate atherosclerotic. Recently, several groups reported that targeting to FCs could effectively ameliorate atherosclerotic (9, 10, 13, 14). Whole-cell vaccines have undergone decades of investigation and exerted powerful antitumor effects (15, 16). Here, we investigated whether the whole-cell vaccination with FCs could be used as novel prevention and treatment strategies to battle atherosclerosis. Like other whole-cell vaccines (15, 17), theoretically the whole FCs could provide some unknown but effective immunodominant epitopes. Those epitopes could be effectively targeted by the immune systems to enhance the clearance of FCs and reduce the accumulation of FCs in plaque.

In the present study, we evaluated a new way to target FCs by employing the whole FCs immunization in ApoE^−/−^ mice. We investigated its efficiency and possible mechanism on reduction of FCs accumulation and attenuation the progression of atherosclerosis. FCs were prepared with peritoneal macrophages by uptake of oxLDL-derived cholesterol. FCs in Freund's adjuvant were then subjected to subcutaneous injection in ApoE^−/−^ mice with developing or established atherosclerosis. To our knowledge, we show that the whole foam cell vaccination is highly effective in reducing plaque size and enhancing plaque stability in atherosclerosis for the first time.

## Materials and Methods

### Animals

Six-week-old male ApoE^−/−^ mice on C57BL/6 background were purchased from Vital River Laboratory Animal Technology Co., Ltd (Beijing, China) and fed with normal chow diet until the dietary intervention. Established atherosclerotic model was induced using 8-week-old male ApoE^−/−^ mice by feeding a Western-type diet (WTD) (18, 19) for therapeutic study. To obtain peritoneal macrophages, 7–8 weeks old male C57BL/6 mice were used. For the preventative study, male ApoE^−/−^ mice were treated with three subcutaneous injections of phosphate buffer saline (PBS, *n* = 8), 0.5 million peritoneal macrophages (Macrophages, *n* = 8) or 0.5 million FCs (FCs, *n* = 8) with combination of Freund's adjuvant every other week from 8 weeks old. These mice were fed a WTD at 11 weeks for the subsequent 16 weeks (20). For the therapeutic study, the male mice were fed a WTD for 12 weeks from 8 weeks old to establish atherosclerosis. The mice with established atherosclerosis were then received three subcutaneous injections of PBS, 0.5 million peritoneal macrophages, or 0.5 million FCs (*n* = 6 per group) emulsified with Freund's adjuvant every other week, and were fed a normal chow diet for an additional 12 weeks. In the two studies, the initial immunization was performed with complete Freund's adjuvant (CFA), followed by two booster injections containing incomplete Freund's adjuvant (IFA). All animal experiments were approved and supervised by the State Key Laboratory of Biotherapy Animal Care and Use Committee (Sichuan University, Chengdu, Sichuan, China).

### Preparation of the Whole-Cell Vaccine

The peritoneum-derived macrophages were isolated from C57BL/6 mice and purified as described previously (21) with some modification. In brief, peritoneal macrophages of C57BL/6 mice were harvested by sterile lavage with cold cell culture medium. Cells were immediately pooled and cultured in RPMI1640 supplemented with 10% fatal bovine serum (GBICO, Australia). After 2–3 h of incubation, non-adherent cells were removed by gentle washing with cell culture medium. The adherent cells were incubated for 24 h and subsequently used in further experiments. To achieve the maximal oxLDL uptake, the macrophages were stimulated with LPS for 12 h and then treated in duplicate by different concentrations of oxLDL from 20 to 100 μg/mL. After 24 h of incubation, the cells were fixed with 3% formalin and stained using Oil Red O and hematoxylin. Six random fields per condition were captured with OLYMPUS BX53 microscope and quantification was performed with Image Pro Plus software.

To confirm the purity of macrophages or FCs, macrophages treated with or without 50 μg/mL oxLDL were stained with APC anti-mouse F4/80 (Biolegend). The analysis was carried out using a BD FACS. In brief, cells were captured via high forward scatter (FSC) and high side scatter (SSC). Favorite cells were gated as shown in Region 1 (R1), of which APC positive cells were selected. Results were expressed in percentage of positive cells. The experiments were performed in triplicate. To prepare the whole-cell vaccine, peritoneal macrophages were incubated with or without 50 μg/mL oxLDL (Guangzhou Yiyuan Biotech. Co, Ltd) for 24 h. After washed three times with sterile PBS, the oxLDL-treated or untreated macrophages were inactivated by formalin. The inactivated cells were finally washed three times and emulsified with equal volume of Freund's adjuvant by grinding water phase and Freund's adjuvant to obtain a water-in-oil emulsion as the whole-cell vaccine (0.5 million cells in 100 μL PBS suspended in an equal volume of Freund's adjuvant per mouse).

### Histological Analysis

At the end of the experiment, mice were sacrificed by cervical dislocation. The mice were immediately bled by cardiac puncture and perfused with cold PBS. Aortas were collected free of connective tissue and fat from the base of ascending aorta to the iliac bifurcation. The hearts were sectioned in the middle, and the upper part was immediately frozen in optimum cut temperature medium in a plastic tube at −80°C. Beginning at the first appearance of the tri-leaflet aortic valve, successive 5 μm transverse sections were made for a distance of 100 μm.

Evaluation of en face lesion formation were performed according to previous reports (10, 22, 23). Briefly, the entire aortas were further cleaned from residual adventitia on a tissue culture dish filled with cold PBS using ophthalmic scissors and tweezers through an optical microscope. The aortas were then cut longitudinally, washed in distilled water. The aortas were dipped in 60% isopropanol briefly, and then stained for 2–3 h in 0.3% Oil Red O dissolved in 60% isopropanol. Next, the aortas were then dipped in 60% isopropanol briefly, washed in distilled water and pinned out on a slide glass. Finally, the cover slides were mounted with distilled water. Lipids are stained red. Images were captured with Nikon SMZ800 microscope. Stained area and total aortic areas were quantified blinded by microscopy and computer aided morphometry. The plaque load was expressed as percentage of the total surface of the aorta according to previous reports (24, 25).

To determine the plaque load and composition, 3~4 sections of the aortic root per mouse were stained with hematoxylin and eosin (22). Corresponding sections were stained with antibody against Mac-2 (10) (Biolegend), MCP-1 (Abcam) or stained for collagen fibers using the Masson's trichrome method. To confirm the presence of FCs, serial sections of the aortic root were stained with hematoxylin and eosin, a macrophages-specific marker (Mac-2) and Oil Red O and hematoxylin, respectively. All images were acquired using the OLYMPUS BX53 microscope. Quantitative analysis of staining was performed blinded by two observers with Image Pro Plus software. In brief, the average plaque area in aortic root of each mouse was computed, and the average group plaque area was quantified for the Control, Macrophages, and FCs group, respectively. Areas of the collagen fibers were determined in trichrome–stained sections and the FCs were identified as the Mac-2 positive part of the plaque. The percentage of FCs or collagen in the lesions was determined by dividing the Mac-2-positive or collagen-positive area by the total lesion surface area. Data are presented as a percentage of total plaque area as reported previously (6, 26–28).

### Antibody Detection

At sacrifice, blood was harvested to obtain plasma to determine the humoral immune response. To prepare oxLDL-coated ELISA plate, oxLDL (10 μg/mL) from the same batch was added into ELISA plate and incubated overnight at 4°C. To prepare macrophages-coated ELISA plate, the peritoneal macrophages (2 × 10^4^ per well) were seeded in ELISA plate. To prepare FCs-coated ELISA plate, the above macrophages-coated ELISA plate was further treated by 50 μg/mL of oxLDL for additional 24 h. All coated plates were washed 3 times with PBS containing 0.05% Tween-20 and thereafter blocked with 2% BSA in PBS for 90 min at room temperature. ELISA plates were then incubated with mouse plasma diluted in 1% BSA for 2 h. After washing, plates were incubated with HRP-labeled anti-mouse IgG, IgG1 or IgG2a (Southern Biotech) at a 1:5000 dilution for 1 h at room temperature. The substrate TMB (100 μL) was then added into the plates for 15 min after washing. Fifty microliter stop solution was finally added and the absorbance was immediately read at 450 nm. Individual plasma from each mouse was tested in duplicate at increasing dilutions (from 1: 100 to 1: 6400) to measure corresponding immunoglobulin IgG. The isotypes of the immunoglobulin were determined at 1: 800 dilution (20). Values were calculated after subtraction of background absorbance. Immunocytochemical analysis (29) was further performed to examine the plasma antibody specificity using PBS-immune, Macrophages-immune or FCs-immune plasma. In addition, whether antibodies stimulated by the whole cell vaccines differed between those expressing ApoE or not was also investigated using cells from ApoE^−/−^ mice. The images were captured and the quantitative analysis of staining was performed with Image Pro Plus software.

### Cellular Immune Response Assay

To explore the cellular immune responses to FCs vaccine, mice immunized with FCs, Macrophages, or PBS emulsified with Freund's adjuvant were killed and spleens were harvested. Spleens were smashed through a 70 mm filter to collect splenocytes. Red blood cells were removed using red blood cell lysing buffer. Spleen cells collected from PBS, Macrophages, or FCs immunized mice were stained for cell surface markers, including CD4 and CD8. Moreover, splenocytes effector cells stained with Carboxyfluorescein Succinimidyl Ester (CFSE) at 2 × 10^5^ cells/well were co-cultured with macrophages or FCs at 10:1 ratio for 72 h and stained for CD3 to visualize cell proliferation (due to dilution of CFSE dye in each cell division, highly proliferating cells are toward the left and non-proliferating cells are toward the right).

### Effect of Immune Plasma on FCs Formation

For the FCs formation inhibition study, the peritoneal macrophages were seeded at 10 × 10^4^/well in 24-well plates and incubated for 24 h. Cells were further cultured for 15 h before experiments in the same conditions but without fatal bovine serum. The macrophages were then incubated in duplicate with or without plasma (50 μL/mL) from FC-immune ApoE^−/−^ mice for 3 h in the presence of DiI-oxLDL (30 μg/mL). To further elaborate the effect of immune plasma on FCs formation, the macrophages were incubated in duplicate with or without plasma (50 μL/mL) from FC-immune ApoE^−/−^ mice for 1 h. After washed with sterile PBS, the cells were incubated with DiI-oxLDL (30 μg/mL) for 3 h. Finally, the cells were washed, fixed with 3% formaldehyde, and stained with Hochest33258 (1 μg/mL). Six random fields per condition were captured by High Content Screening. Internalized oxLDL was revealed by DiI fluorescence and analysis was performed with Image Pro Plus software (30).

### Analysis of Plasma Lipids and Cytokines

Retro-orbital blood was obtained via EDTA-coated micro capillary tubes during organ harvest at the end of the study. The whole blood of mice after immunization had been analyzed by Celltac F automated hematology analyzer to investigate the blood components, including leukocytes, erythrocytes, thrombocytes, lymphocytes, monocytes, and neutrophils. The remaining whole blood was spun at 10,000 rpm for 10 min at 4°C. The supernatant was collected and frozen at −20°C until analysis to reduce multiple freeze/thaw cycles. No-fasting plasma lipids, including total cholesterol, LDL cholesterol, HDL cholesterol, and triglycerides levels, were determined using plasma biochemistry automatic analyzer (Hitachi High-Technologies Corp., Minato-ku, Tokyo, Japan) as described previously (31, 32). Mouse IFN-γ ELISA Kit, Mouse IL-6 ELISA Kit, and Mouse MCP-1 ELISA Set were purchased from BD Pharmingen. Mouse TGF-β1 Precoated ELISA kit were provided by Boster Biological Technology Co., Ltd. The levels of IFN-γ, IL-6, MCP-1, and TGF-β1 were determined according to the manufacturer's protocol after the plasma samples being diluted at 1:2.

### Statistical Analysis

All values are presented as mean ± SEM unless specified. Data of multiple groups were analyzed using a non-parametric one-way ANOVA, followed by the Bonferroni's post-test. Statistical analysis was performed using Graphpad Prism 5.0. The *p*-values < 0.05 were considered significant.

## Results

### FCs Preparation

**Figure 1:**
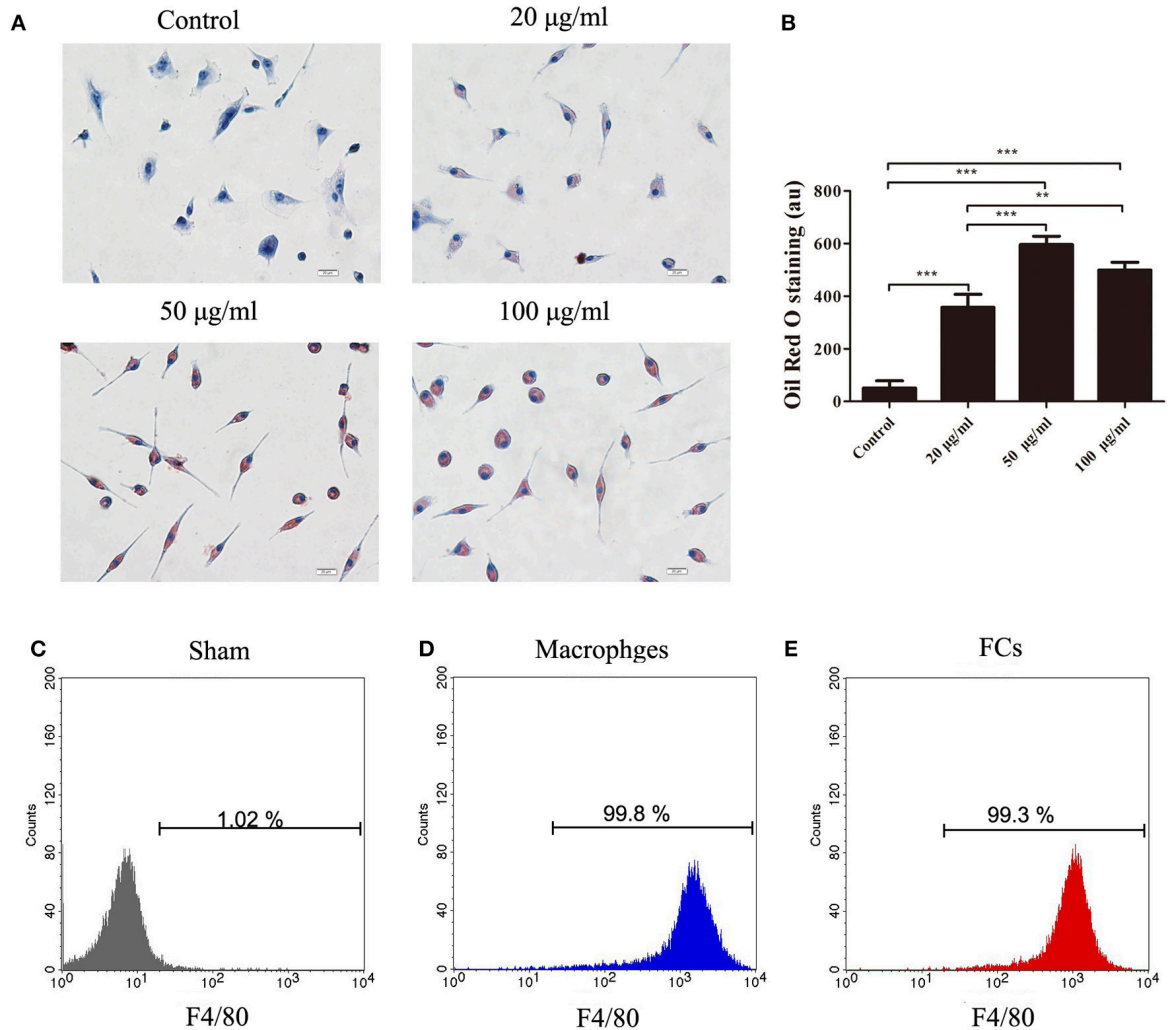
The oxLDL uptake by peritoneal macrophages treated with or without oxLDL (20, 50 or 100 μg/mL) for 24 h. Internalized oxLDL were stained with Oil Red O. Six random fields per condition were observed with OLYMPUS BX53 microscope. **(A)** A representative image is shown for each condition. Scale bars, 20 μm. **(B)** Quantification of intracellular oxLDL. The mean ± SEM of Oil Red O staining per condition is expressed in arbitrary units (au). Peritoneal macrophages or FCs were stained with APC anti-mouse F4/80. Flow cytometry histograms for macrophages **(D)**, and FCs **(E)**, respectively. Unlabeled macrophages were used as negative controls **(C)**. The experiment was performed in triplicate. ^**^p < 0.01; ^***^p < 0.001.

To prepare FCs, the isolated peritoneal macrophages were cultured with increasing concentrations of oxLDL. 50 μg/mL oxLDL was sufficient to lead to the maximal oxLDL uptake by macrophages. Further increase of oxLDL concentration to 100 μg/mL enhanced no significant uptake of oxLDL by macrophages (Figures 1A,B). The purity of macrophages and FCs was confirmed. More than 99% cells were identified as F4/80-positive cells by flow cytometry (Figures 1C–E). Additionally, there was no significant difference about FSChi and SSChi between peritoneal macrophages treated with or without oxLDL (50 μg/mL) for 24 h (Figure S1). Hence, we chose this condition to generate FCs for further experiments.

### FCs Treatment Diminish Initial Atherosclerotic Lesion

**Figure 2:**
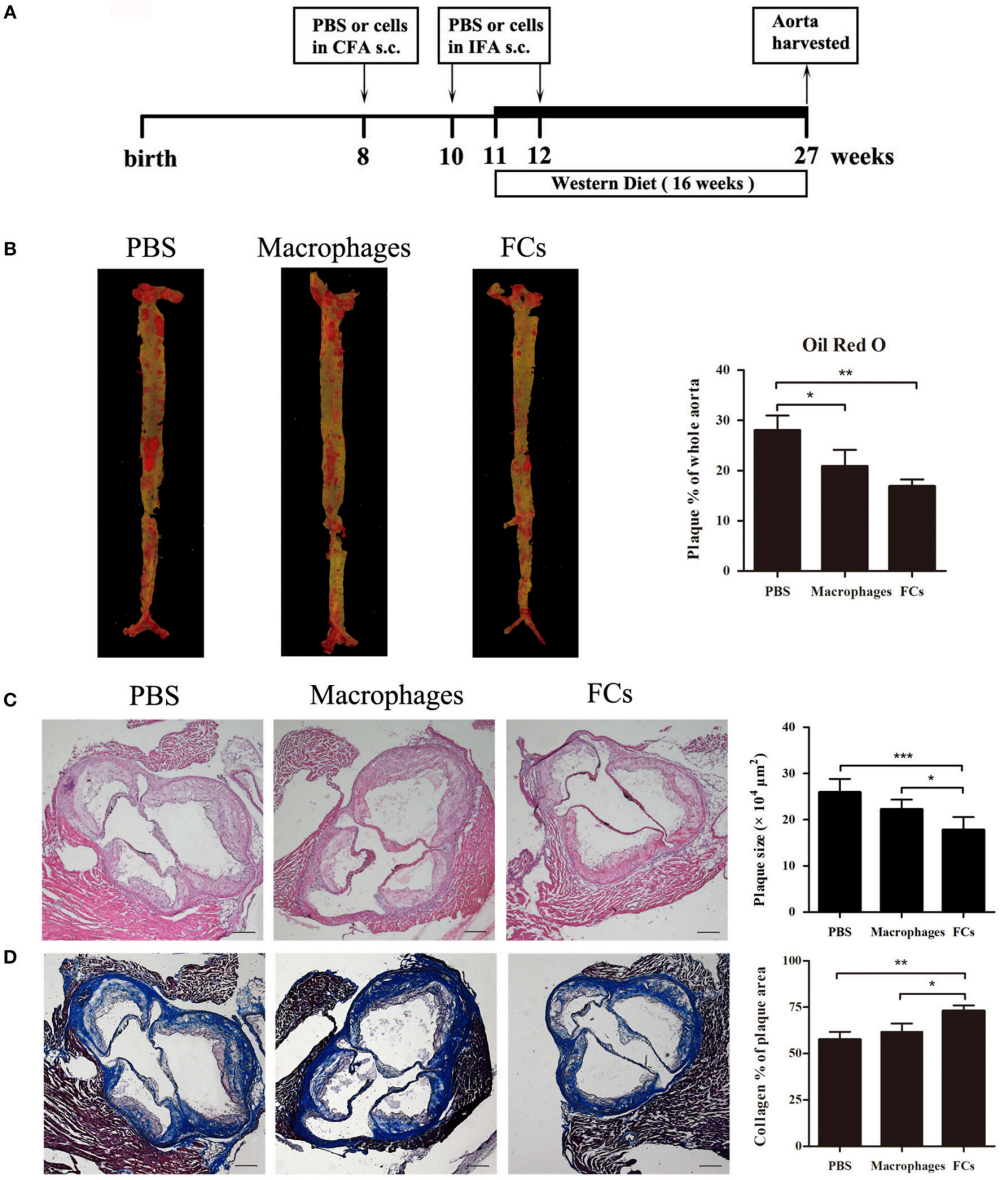
FCs immunization attenuates initial atherosclerotic lesion. **(A)** Schematic illustration of the prevention experimental setup. **(B)** Evaluation of en face lesion size of the whole aorta was carried out by Oil Red O staining. **(C)** Plaque size in the three-valve area of the aortic root was assessed by hematoxylin and eosin staining. **(D)** Collagen content in the aortic root was determined by Masson's Trichrome staining as the percentage of the total lesion area. Scale bars, 200 μm. All data are presented as mean ± SEM and are representative of all mice. *n* = 5–6 in each group; ^*^p < 0.05; ^**^p < 0.01; ^***^p < 0.001.

For the preventative study, the ApoE^−/−^ mice were immunized with the prepared FCs vaccine through subcutaneous injections according to the schematic illustration (Figure 2A). At the end of the experiment, we found significant reduction of 40% in FC-treated mice and 26% in macrophages-treated mice in en face lesion size by Oil Red O staining of whole aortas when compared with PBS immunized mice (Figure 2B). The lesion sizes in the three-valve area of the aortic root were further analyzed by hematoxylin and eosin staining. A dramatic 31% decrease was noted in FCs-immune mice, whereas only 14% decrease was observed in macrophages-immune mice (Figure 2C). Furthermore, we observed a more stable lesion phenotype in FC-treated mice. The aortic root lesions of FC-treated were composed of 72% collagen, whereas those in control mice were only 57% collagen (Figure 2D).

### FCs Vaccine Is Beneficial for Plaque Stabilization in Established Atherosclerosis

**Figure 3:**
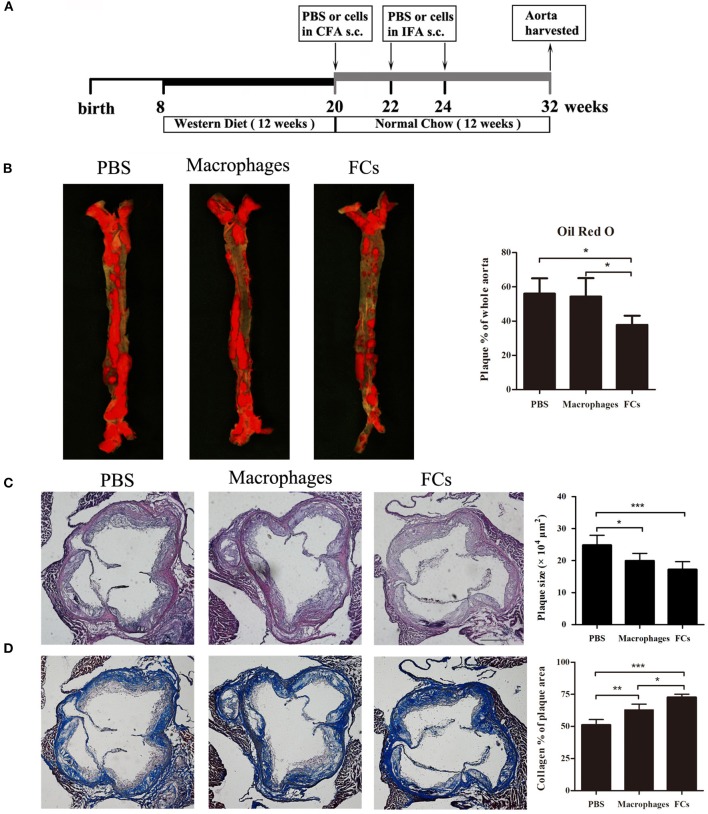
FCs vaccination attenuates advanced lesion. **(A)** Experimental design of the therapeutic study. **(B)** En face lesion size of the whole aorta. **(C)** Plaque size in the three-valve area of the aortic root. **(D)** Collagen content in the aortic root. Scale bars, 200 μm. All data are presented as mean ± SEM and are representative of all mice. *n* = 5–6 in each group; ^*^p < 0.05; ^**^p < 0.01; ^***^p < 0.001.

For the therapeutic study, the ApoE^−/−^ mice were treated with FCs vaccine according to the experimental design (Figure 3A). Eight weeks after the last immunization of FCs vaccine, the size of atherosclerotic lesions in the whole aorta greatly decreased by 33% through the en face staining analysis of Oil Red O (Figure 3B). In contrast, macrophages-immune mice had no significant change in the lesion size by comparison with control. The plaque load in aortic root was significantly decreased about 20% in macrophages-immune mice and 31% in FCs-immune mice, respectively, (Figure 3C). Additionally, collagen content in aortic root lesion of FCs treated mice significantly increased by about 42% and those macrophages-treated mice increased by about 23% compared with PBS treated group. Of note, FCs immunization induced a more stable plaque when compared with macrophages vaccination (Figure 3D).

### FC Immunization Reduces the Accumulation of FCs in Plaque

**Figure 4:**
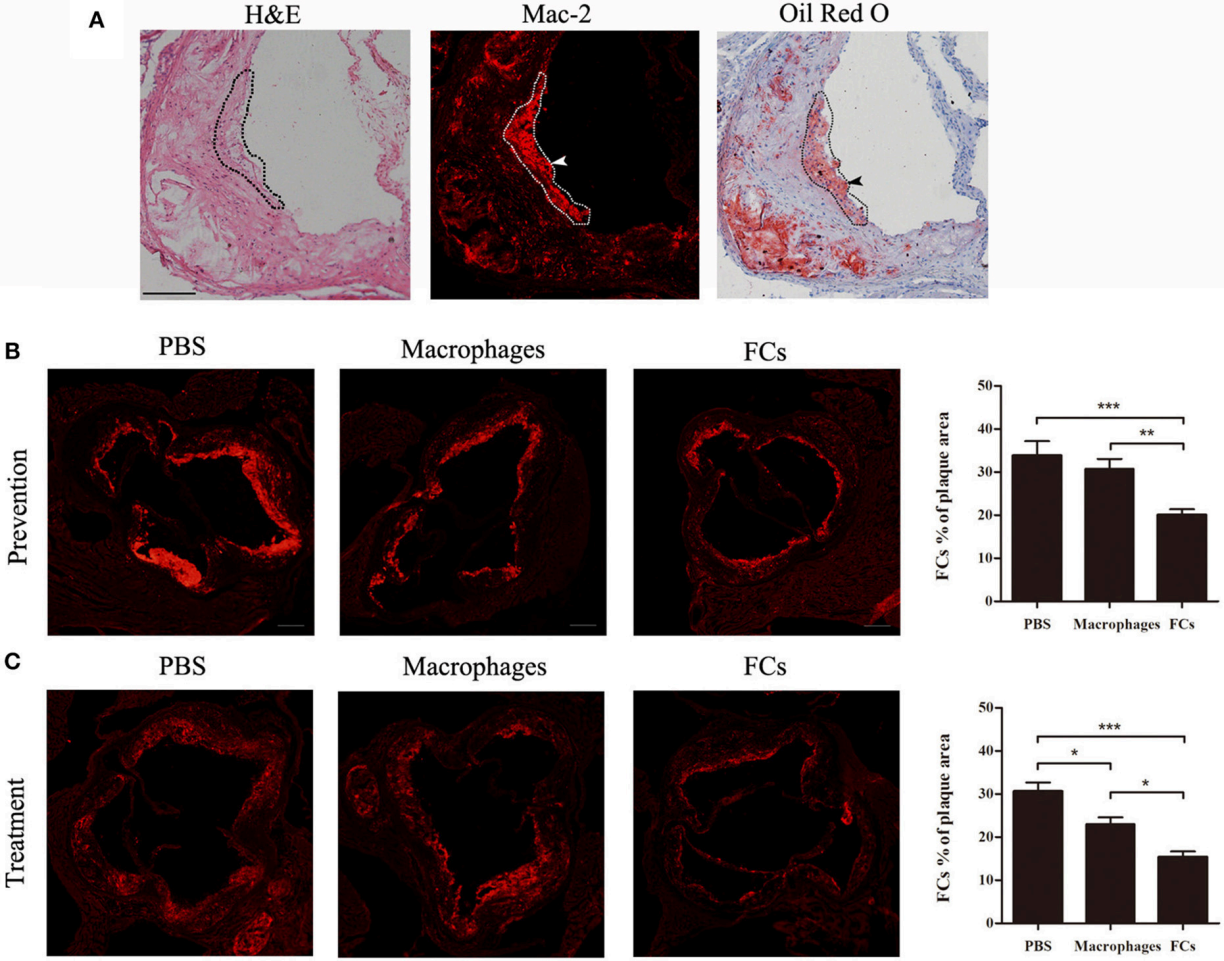
FCs vaccine decreases accumulation of FCs in plaque. **(A)** Serial cryosections of the aortic root were stained with hematoxylin and eosin, Mac-2, and Oil Red O, and hematoxylin, respectively. Representative photographs of corresponding staining were shown with co-location of Mac-2 and Oil Red O positive area outlined and FCs depicted by arrows. Scale bars, 100 μm. The FCs content in the aortic root of initial **(B)** and advanced **(C)** plaque were evaluated as the Mac-2 positive area per plaque area. Scale bars, 200 μm. ^*^p < 0.05; ^**^p < 0.01; ^***^p < 0.001.

To confirm the presence of FCs in plaque, serial cryosections of the aortic root were stained with H&E, a macrophages-specific marker (Mac-2) and Oil Red O and hematoxylin. Co-localization of Mac-2 and Oil Red O positive area was obvious and marked by dotted lines. A representative Mac-2 and Oil Red O dual-positive cell was shown with arrowheads (Figure 4A). These data argued that Mac-2 positive area could represent FCs distribution in atherosclerotic plaque. Next, we investigated whether immunization could reduce the accumulation of FCs in atherosclerotic plaque. As shown in Figures 4B,C, FCs-immunization significantly reduced FCs accumulation in the initial and advanced plaque (40.7 and 49.9%, respectively), whereas macrophages-immunization demonstrated only a modest decrease (9.6 and 24.8%, respectively) when compared with PBS control.

### FCs Vaccination Induces Humoral and Cellular Responses

**Figure 5:**
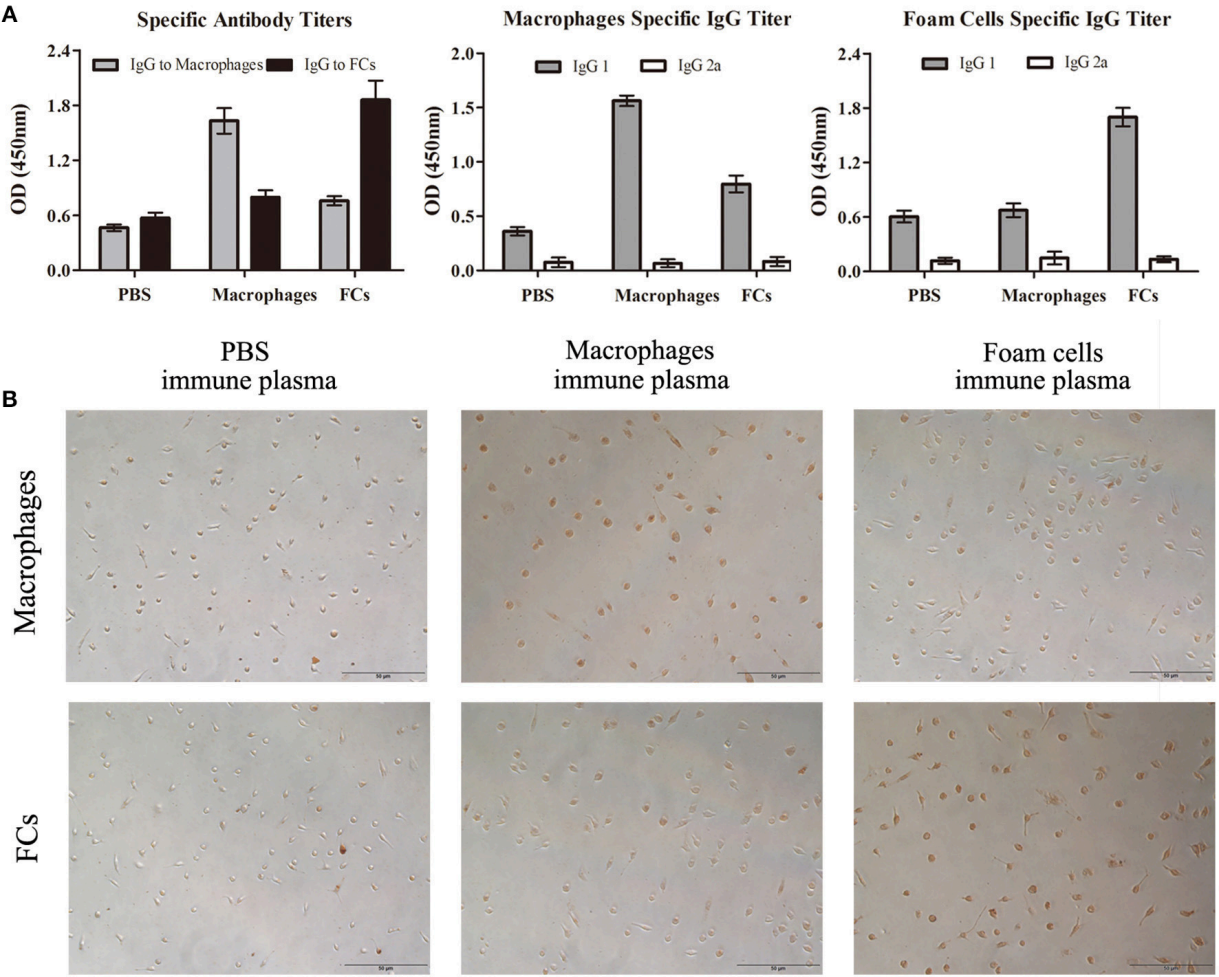
FCs immunization results in a specific antibody response in initial atherosclerosis. **(A)** ELISA was performed to measure the specific antibodies to FCs and macrophages as well as immunoglobulin isotypes including IgG1 and IgG2a titers. Data are shown at plasma dilutions of 1: 800 for each group (*n* = 8). OD, optical density. **(B)** Peritoneal macrophages and FCs were fixed and stained with PBS-immune, Macrophages-immune or FCs-immune plasma (dilution 1:50). Scale bars, 50 μm.

To explore the humoral response to the vaccination, antibodies in mice plasma generated by immunization were determined. ELISA studies demonstrated that the subcutaneous immunization of FCs in Freund's adjuvant induced a significant elevated titer of IgG against FCs compared with other groups (Figure 5A). Of note, there was no significant difference about anti-oxLDL titers among three groups at different dilutions (Figure S2). These data confirmed the specificity of the antibodies stimulated by FCs vaccine. In addition, the antibodies induced by FCs or normal macrophages were almost exclusively of the IgG1 isotype indicative of strong T helper type 2 immune responses (Figure 5A).

To further investigate the specificity of the antibodies stimulated by FCs vaccine, FCs and peritoneal macrophages were stained with PBS-immune, Macrophages-immune, or FCs-immune mouse plasma, respectively. The immunostaining images showed that the immune plasma from FC-vaccinated mice could recognize and bind to FCs but had lower reactivity to normal macrophages (Figure 5B), which validated the ELISA results. Additionally, FCs-immune mouse plasma could recognize and bind to FCs from ApoE^−/−^ mice (Figure S3), suggesting that there is no significant difference in the antibody response to FCs between ApoE expressing or not.

**Figure 6:**
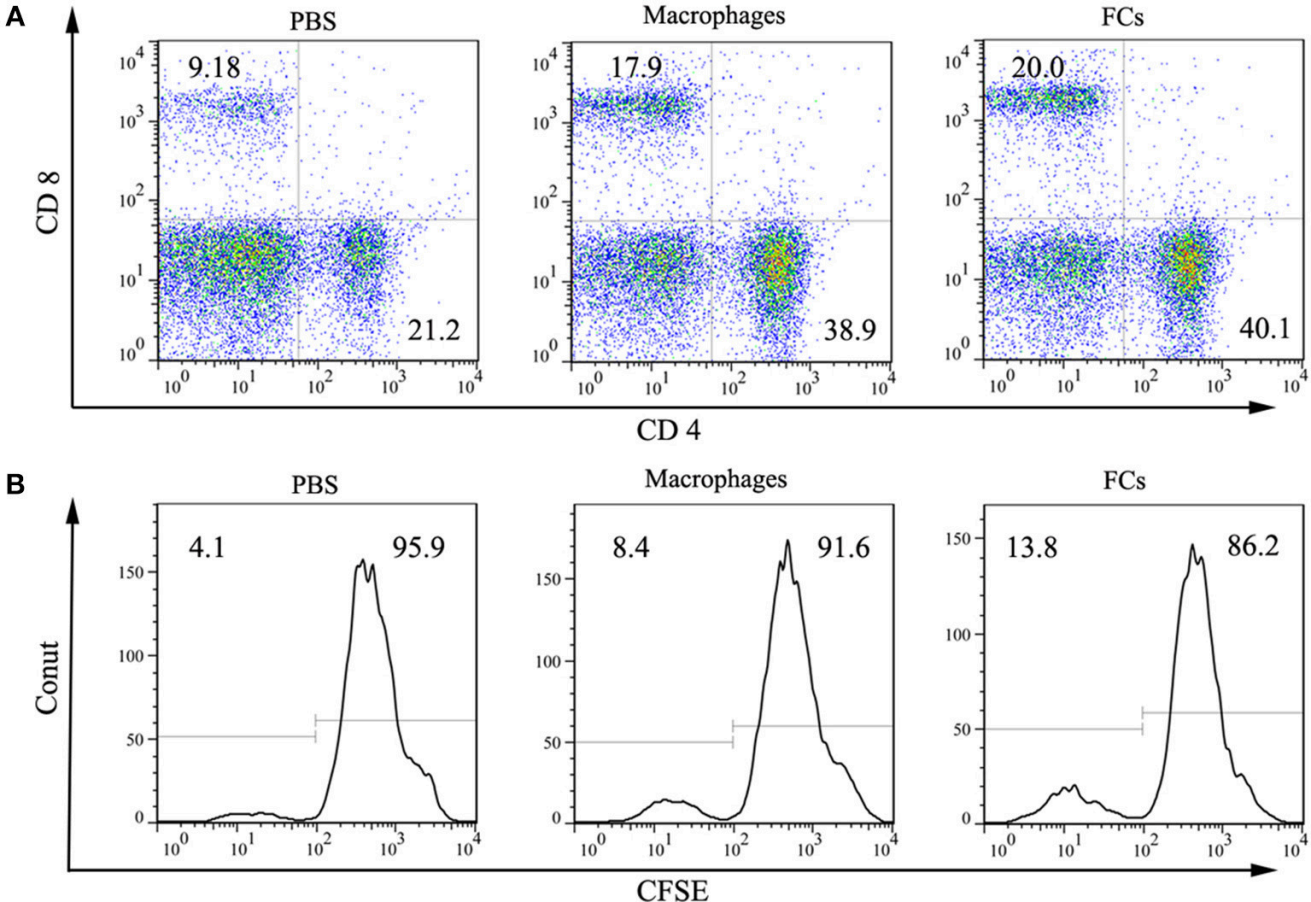
FCs immunization induces cellar response. **(A)** Macrophages and FCs vaccines significantly increased CD4^+^ T cell and CD8^+^ T cell populations in spleen compared with those PBS immunized mice. **(B)** Total splenocytes were cultured *in vitro* in the presence of FCs or Macrophages. Proliferation of CD3 positive T cell was measured by CFSE with peaks reflecting cell divisions.

Cellular responses on FCs immunization were also investigated. Both macrophages and FCs immunization significantly increased the CD4^+^ and CD8^+^ T lymphocytes levels in spleen (Figure 6A). Moreover, incubation splenocytes from FCs immunized mice with FCs could induce an overt T cell proliferation, suggesting that an obvious cellular immune response was raised by immunization (Figure 6B).

### Immune Plasma Blocks the Process of FCs Formation

**Figure 7:**
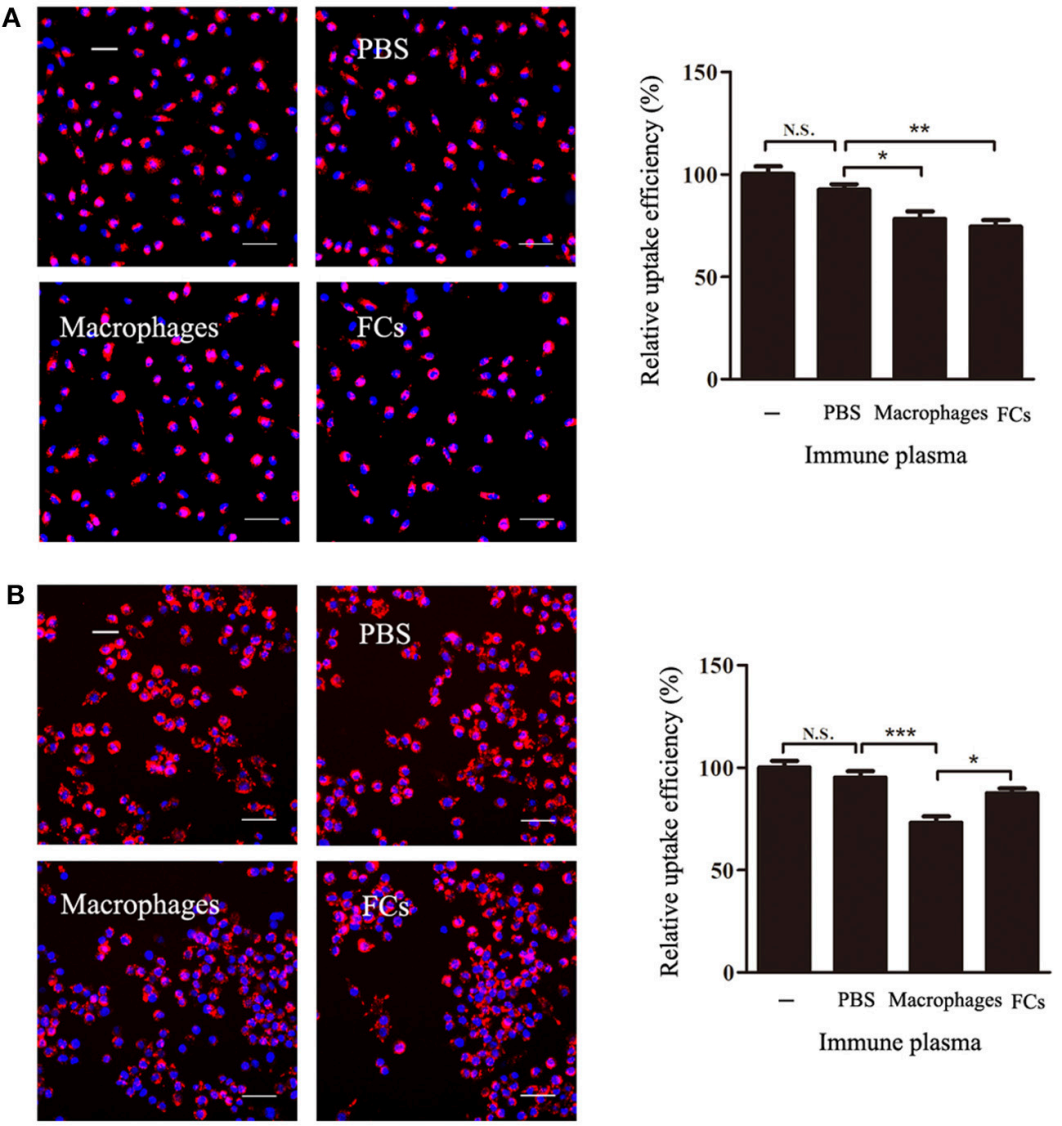
FCs immune plasma blocks the process of FCs formation. **(A)** For the FCs formation inhibition study, the macrophages were incubated in duplicate with or without plasma (50 μL/mL) from FC-immune ApoE^−/−^ mice for 3 h in the presence of DiI-oxLDL. **(B)** Further investigation of the effect of immune plasma on FCs formation. Macrophages were treated with or without plasma (50 μL/mL) from PBS-immune, macrophages-immune, or FC-immune ApoE^−/−^ mice for 1 h. After washed, cells were cultured with 30 μg/mL DiI-oxLDL for 3 h **(B)**. Internalized oxLDL was revealed by DiI fluorescence, and six random fields per condition were captured by High Content Screening. A representative image is shown for each condition. Scale bars, 50 μm. ^*^p < 0.05; ^**^p < 0.01; ^***^p < 0.001.

To test whether the FCs-immune plasma could block the process of FCs formation, FCs-immune plasma was incubated with macrophages in the presence or absence of oxLDL. The presence of FC-immune mouse plasma during macrophages incubation with oxLDL resulted in a significant decrease by about 20% of the intracellular oxLDL (Figure 7A). However, when the peritoneal macrophages were pre-exposed to immune plasma, none significant decrease of oxLDL uptake by FCs-immune plasma treatment was observed. In contrast, the oxLDL uptake was markedly reduced by macrophages-immune plasma treatment in comparison with PBS-immune plasma treatment (Figure 7B).

### FCs Vaccine Significantly Modulates Cytokine Production *in vivo*

**Figure 8:**
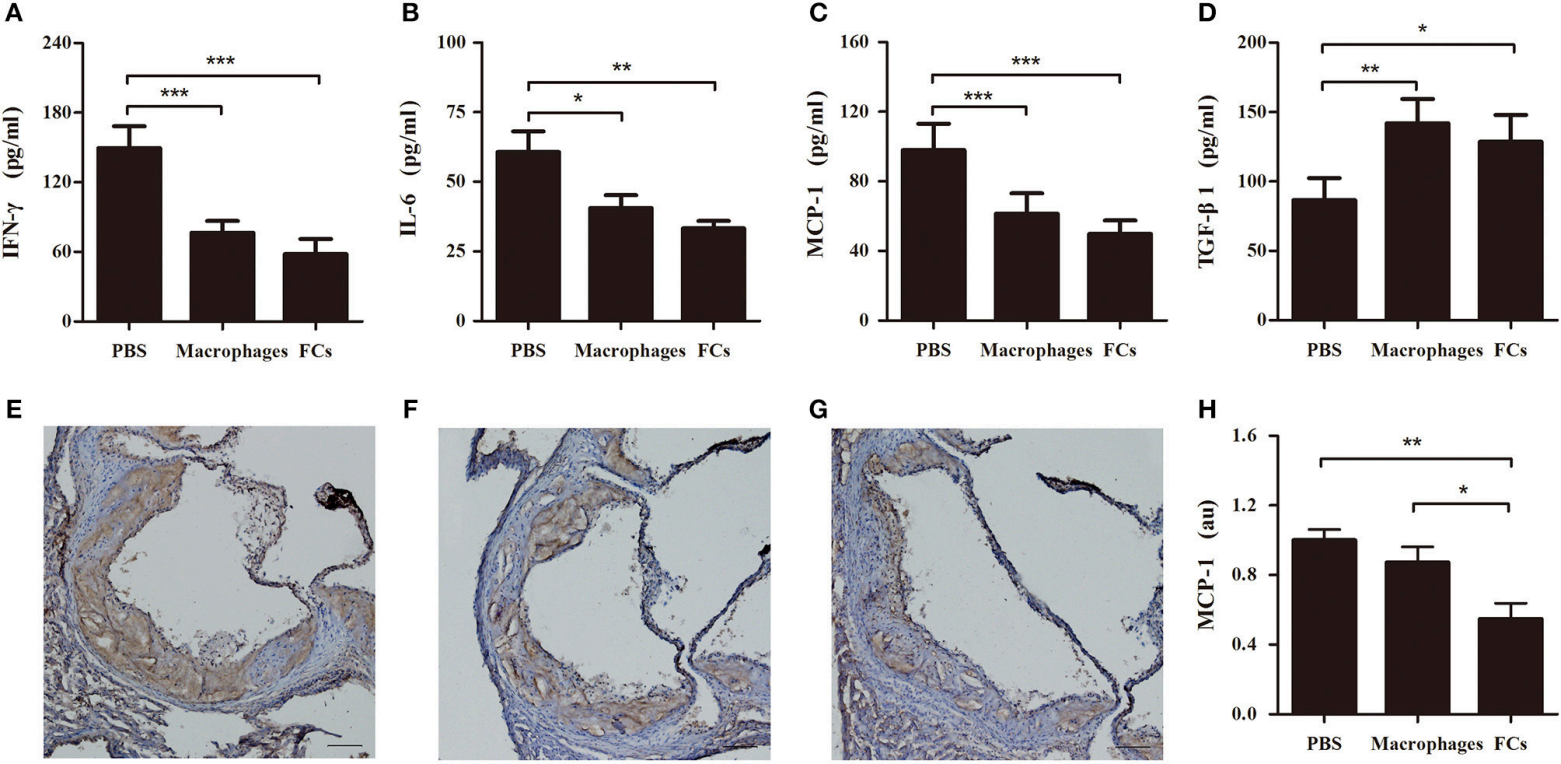
FCs vaccination modulates cytokines in initial atherosclerosis. The levels of IFN-γ **(A)**, IL-6 **(B)**, MCP-1 **(C)**, and TGF-β1 **(D)** in immune ApoE^−/−^ mouse plasma were determined at 1:2 dilution by ELISA according to the manufacturer's protocol. *n* = 8 in each group. Aortic roots were sectioned and stained with MCP-1. Representative photographs of MCP-1 immunostaining of PBS **(E)**, macrophages **(F)**, and FCs **(G)** immunized mice, respectively. Scale bars, 100 μm. **(H)** Quantification of MCP-1 expression in atherosclerotic lesion area of aortic root. *n* = 6 in each group; ^*^p < 0.05; ^**^p < 0.01; ^***^p < 0.001.

Plasma levels of proinflammatory cytokine IFN-γ and IL-6 were significantly reduced by immunization with FCs compared to the control (Figures 8A,B). A similar trend was observed in plasma levels of proatherogenic chemokine MCP-1 (Figure 8C). Consistent with the change of plasma MCP-1 level, FCs immunization also reduced MCP-1 expression (brown stain) in the initial atherosclerotic lesions (Figures 8E–H). Moreover, FCs treatment dramatically increased the plasma levels of atheroprotective cytokine TGF-β1 (Figure 8D). In addition, no significant effects of macrophages or FCs immunization on body weight, plasma lipid profile and blood cells were observed among immune groups (Table 1).

**Table 1:** The effects of immunization on body weight, plasma lipid profile and blood cells of ApoE^−/−^ mice.

	**PBS**	**Macrophages**	**FCs**
Weight (g)	23.8 ± 4.1	26.9 ± 3.2	24 ± 1.0
TC (mmol/L)	18.83 ± 3.14	22.48 ± 1.53	19.8 ± 2.68
TG (mmol/L)	1.96 ± 0.59	1.99 ± 0.92	1.56 ± 0.39
HDL-C (mmol/L)	0.22 ± 0.04	0.18 ± 0.07	0.2 ± 0.05
LDL-C (mmol/L)	1.54 ± 0.74	3.77 ± 1.52	2.43 ± 0.90
Leukocyte 10^9^/L	6.5 ± 1.7	4.6 ± 1.3	5.4 ± 1.9
Erythrocyte 10^12^/L	7.8 ± 1.1	8.6 ± 1.2	8.2 ± 1.6
Thrombocyte 10^9^/L	294 ± 85	210 ± 83	206 ± 46
Lymphocyte 10^9^/L	1.91 ± 0.48	1.86 ± 0.30	1.72 ± 0.53
Monocyte 10^9^/L	0.75 ± 0.26	0.67 ± 0.15	0.69 ± 0.25
Neutrophils 10^9^/L	3.2 ± 1.0	2.3 ± 0.8	2.9 ± 1.4

*TC indicates total cholesterol; TG, Triglycerides; HDL-C, high-density lipoprotein cholesterol; LDL-C, low-density lipoprotein cholesterol; Values are presented as mean ± SD, n = 6/group*.

## Discussion

Atherosclerosis is an immune-mediated inflammatory disease of the arterial wall. Both the innate and adaptive immune systems responding to many endogenous and exogenous antigens. Hence, it is conceivable that an immunomodulatory strategy via active immunization against many of these antigens could potentially alter the natural history of atherosclerosis (33). Recently, modulation of the immune response against atherosclerotic plaque antigen(s) has attracted attention, including oxLDL (34), apolipoprotein B peptides (23, 24, 35) and heat shock protein (36). LDL is a large, heterogeneous molecule containing a diverse cargo of apolipoproteins, cholesteryl esters, triglycerides, and phospholipids. It would be impractical to use whole homologous LDL as an antigen in a clinically-usable vaccine formulation. Actually, immunotherapy based on apoB-100 or oxLDL loaded dendritic cells may be alternative approaches to attenuates atherosclerosis (6, 25, 37). Macrophages took up excessive oxLDL and then transform into FCs and form the fatty streak (38). FCs accumulation in lesion plays a crucial role in the initiation and progression of atherosclerosis (26). Different with those pervious vaccines design, we designed a whole-cell vaccine based on FCs to targeting FCs. We then investigated its efficiency and possible mechanism on preventative and therapeutic effects on atherosclerosis. Whole-cell vaccines have undergone a relative long term of investigation and exerted powerful therapeutic effects by providing multiple and unknown antigens (17, 39, 40). Our results demonstrated that the whole FCs vaccine induced strong immune responses and positively improved atherosclerosis by declining the en face lesion size in whole aorta, reducing plaque load, and FCs accumulation in aortic root, and enhancing the stability of atherosclerotic lesion by raising FCs-specific IgG and modulating cytokine production.

In the present study, FCs used for immunization were prepared with peritoneal macrophages from C57BL/6 mice (donor mice) as reported previously (6, 37). The whole FCs vaccination induced the strong humoral immune responses with the production of FCs-specific polyclonal antibodies. FCs-immune mouse plasma could selectively recognize and bind to FCs. As expected, FCs-immune mouse plasma showed minimal reactivity to normal macrophages or oxLDL, suggesting that the immune polyclonal plasma is independent of normal macrophages or oxLDL. Moreover, our results showed that the most of anti-FCs polyclonal antibodies is IgG1 but not IgG2a. The FCs-specific IgG may exert its beneficial effects possibly through different ways. Like other whole-cell vaccines, FCs immunization raised effective polyclonal antibodies because of multiple and unknown antigens provided by FCs. The FCs-induced polyclonal antibodies might selectively bind to the FCs, and then induce the antibody-mediated immune response and enhance the clearance of FCs in lesion, finally reduce the plaque formation. Incubation splenocytes from FCs immunized mice with FCs induced overt T cell proliferation. Our results combined with those from the previous studies strongly suggest that the reactive T cells might be beneficial to the athero-protection elicited by immunization (20, 37, 41). However, further studies are still required to elucidate the cooperative anti-atherosclerotic effect of the cellular immune response and the humoral immune response.

Interestingly, pretreated macrophages with the FCs immune plasma could not effectively block the phagocytosis of oxLDL. However, incubated with immune plasma in the presence of oxLDL could attenuate the formation of FCs. And also, it has been observed that the FCs immune plasma did not bind to the oxLDL nor normal macrophages. Taken together, it suggested that the FCs-immune plasma significantly inhibit the formation of FCs mainly by targeting to the process of FCs formation but not directly to the normal macrophages nor oxLDL. Nevertheless, more detailed future studies will be needed to identify what the immune plasma targets and uncover how it works. Although, it is difficult to determine the epitopes specific to FCs or even identify their molecule markers different from normal macrophages, further identification of immunodominant epitopes and uniquer molecule markers of FCs is of great interest.

Atherosclerosis is a chronic inflammatory disease involving pro-inflammatory and anti-inflammatory pathologic process (22, 42, 43). Inflammation is not effectively resolved in atherosclerosis (44). Cell cytokines are important factors involved in the progression of atherosclerosis (22, 44, 45). IFN-γ, the pro-inflammatory mediator, promotes foam cell formation (46). IL-6 regulates monocytes to macrophages differentiation and activation in the aorta along with MCP-1 (47). FCs vaccination significantly decreased the circulating levels of IFN-γ, IL-6, and MCP-1 and the expression of MCP-1 in aortic root. Thus, it could be inferred that the anti-atherosclerotic effect of FCs vaccination was partially ascribed to the decrease of pro-inflammatory cytokines and chemotactic factor, which reduced the recruitment of monocytes/macrophages and inflammation in atherosclerosis. TGF-β1 is a potent stimulator of collagen secretion and cause collagen deposition (48). In parallel with increase of circulating TGF-β1, dramatic collagen deposition in aortic root was observed in not only early lesions but also advanced lesions by FCs vaccination. We're surprised to find this interesting phenomenon although some other researchers also observed the similar consistence of collagen deposition in the lesions by different immunization (6, 27). The consistency of collagen deposition in the lesions observed in the field of atherosclerosis immunization partially resulted from adjuvant and the other similarity of different vaccines. FCs immunization stabilized the plaque by increasing collagen content in the aorta root owing to elevated TGF-β1. FCs immunization down-regulated the expression level of atherosclerosis related pro-inflammatory cytokines, including IFN-γ, MCP-1, and IL-6 and enhanced the lesion stability with a significant increase in TGF-β1 level. Dampen inflammation and enhance inflammation resolution were beneficial to the treatment of atherosclerosis (44, 49, 50).

Additionally, macrophages-immune mice also displayed attenuation of atherosclerosis by macrophages-specific antibodies and cytokines modulation, in despite of lower protective efficacy than FCs vaccine. Although macrophages-specific plasma could not selectively recognize the FCs, it can indirectly inhibit the development of atherosclerosis via regulating the expression level of cytokines of the vaccinated mice. However, unlike FCs immunization, the macrophages immunization could not significantly decrease the whole en face lesion area of established atherosclerotic lesion. This difference could be explained by the difference of humoral and cellular immune response raised by vaccination. The antibodies raised by macrophages vaccination might mainly recognize and bind to normal macrophages. Actually, it could not well-recognize and bind to FCs or induce the antibody-mediated immune clearance in the established lesion. In addition, such macrophages vaccine might have serious side effects and cannot be used as anti-atherosclerotic approach because macrophages specific antibodies might affect the normal function of macrophages (51). In the present study, FC-immune mice plasma presented a slight cross binding activity to normal macrophages. No difference about the titers of plasma antibodies specific to oxLDL among these three groups was detected, indicating that the protective effect of FCs immunization was attributed to FCs itself but not macrophages or oxLDL. The low cross-reactivity of FCs-immune plasma with normal macrophages might be caused by the same immunodominant epitopes between FCs and macrophages, because FCs were derived from macrophages (52). These results indicate that during the formation of FCs from macrophages, FCs might generate some unknown and unique immunodominant epitopes. Despite the presence of FCs was confirmed by Mac-2 and Oil Red O dual-positive cell, their molecule markers different from normal macrophages are hardly to identify. Currently, it is difficult to determine the immune epitopes specific to FCs. Of note, because of the significant function difference against atherosclerosis by the FCs and macrophages, it is of great interest to further identify the effective immunodominant epitopes and new antibody targeting FCs using B cell technology. Moreover, this exploration will increase our understanding of the immune atheroprotective mechanism of FCs vaccination.

In conclusion, our results demonstrated that the whole FCs vaccine positively improved atherosclerosis by declining the lesion area, reducing plaque size, and enhancing the stability of atherosclerotic lesion by inducing strong humoral and cellar immune responses. Taken together, these results might provide new insight to find new vaccine and antibodies specifically targeting FCs to conquer atherosclerosis at different stages.

## Author Contributions

YW, ZZ, and FW conceived and designed the experiments. FW, AF, and QJ conducted most of the experiments, performed the statistical analysis, and drafted the manuscript. FW, ZZ, XS, CJ, and JW participated in literature research and manuscript editing. DF helped with animal experiments. YL, CJ, and XT participated in immunohistochemistry and other pathological experiments. All authors reviewed the manuscript.

### Conflict of Interest Statement

The authors declare that the research was conducted in the absence of any commercial or financial relationships that could be construed as a potential conflict of interest.

## Abbreviations

FCs: Macrophage foam cells
WTD: Western-type diet
oxLDL: oxygenized low density lipoprotein
CFA: complete Freund's adjuvant
IFA: incomplete Freund's adjuvant.
