# Supplementary material for: Macrophage Foam Cell-Targeting Immunization Attenuates Atherosclerosis
Source: Front Immunol. 2019 Jan 10;9:3127. doi: 10.3389/fimmu.2018.03127 (PMC6335275; doi:10.3389/fimmu.2018.03127)
Supplement: Supplementary file 1 [file Data_Sheet_1.PDF]

Supplementary Materials

Macrophage foam cell-targeting immunization attenuates atherosclerosis

Fazhan Wang[1†], Zhi Zhang[1,2†], Aiping Fang[1,3], Quansheng Jin[1], Dailong Fang[1], Yongmei Liu[1], Jinhui Wu~~Chunling Jiang~~[1], Xiaoyue Tan[4], Yuquan Wei[1*], Chunling Jiang~~Jinhui Wu~~[1*], Xiangrong Song[1*]

[1] *State Key Laboratory of Biotherapy, Department of Anesthesiology and Translational Neuroscience Center~~Geriatrics and Cancer Center~~, West China Hospital, Sichuan University, and Collaborative Innovation Center for Biotherapy, Sichuan University, Chengdu 610041, China*

[2] *School of Chemical and Pharmaceutical Engineering, Sichuan University of Science and Engineering, Zigong 643000, China*

[3] *West China School of Public Health, Sichuan University, Chengdu 610041, China*

[4] *Department of Pathology/Collaborative Innovation Center of Biotherapy, Medical School of Nankai University, Tianjin 300071, China*

[†] These authors contributed equally to this article.

[*] Corresponding author: Xiangrong Song; Chunling Jiang~~Jinhui Wu~~; Yuquan Wei

Tel.: +86 28 85503817; Fax: +86 28 85503817.

Email: songxr@scu.edu.cn (X.Song); jiang_chunling@yahoo.com (C. Jiang)~~wujinhui@scu.edu.cn (J. Wu)~~; yqwei@scu.edu.cn (Y. Wei)

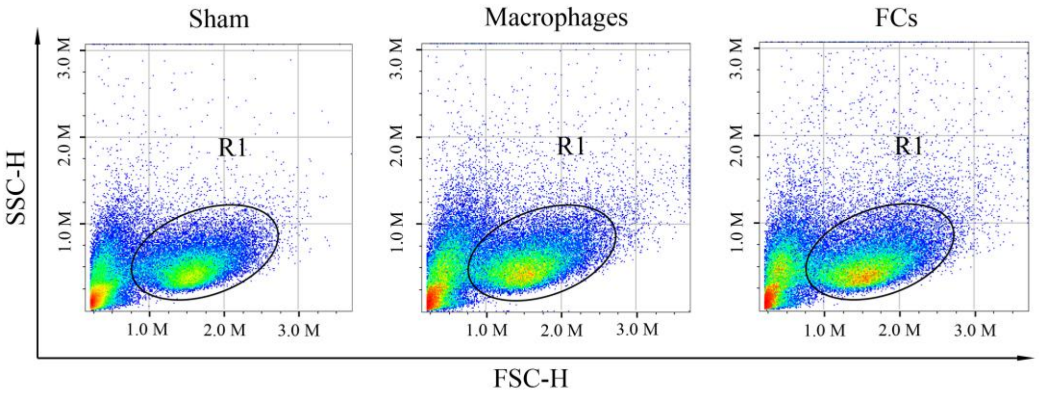

Figure S1 Cells and gating strategies were shown in scatter plots. Cells were captured via high forward scatter (FSC) and high side scatter (SSC). Favorite cells were gated as shown in Region 1 (R1).

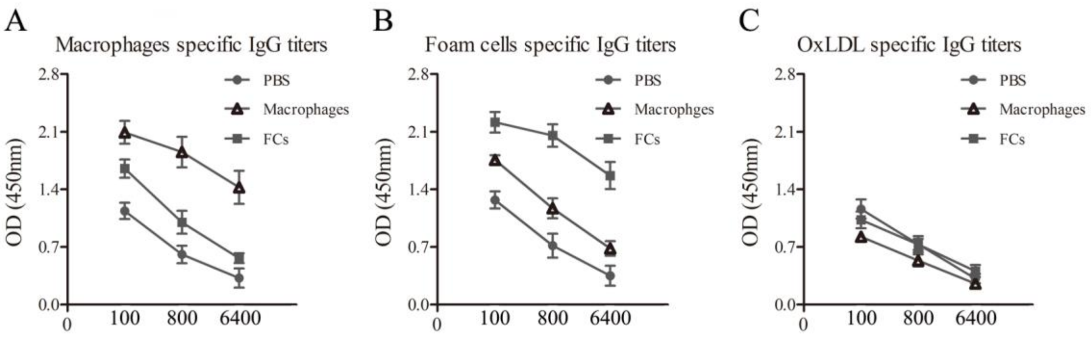

Figure S~~1~~2 FCs immunization induced a specific systemic humoral response in initial atherosclerosis. Plasma from animals immunized with PBS, macrophages or FCs was collected at termination and used to assess the levels of Macrophages (A), FCs (B) and oxLDL (C) IgG synthesis by ELISA. Individual plasma from each mouse was tested at increasing dilutions (from 1:100 to 1:6400). Data are expressed in optical density (OD) units measured at 450 nm and represented as mean ± SEM for each group. n = 8 per group.

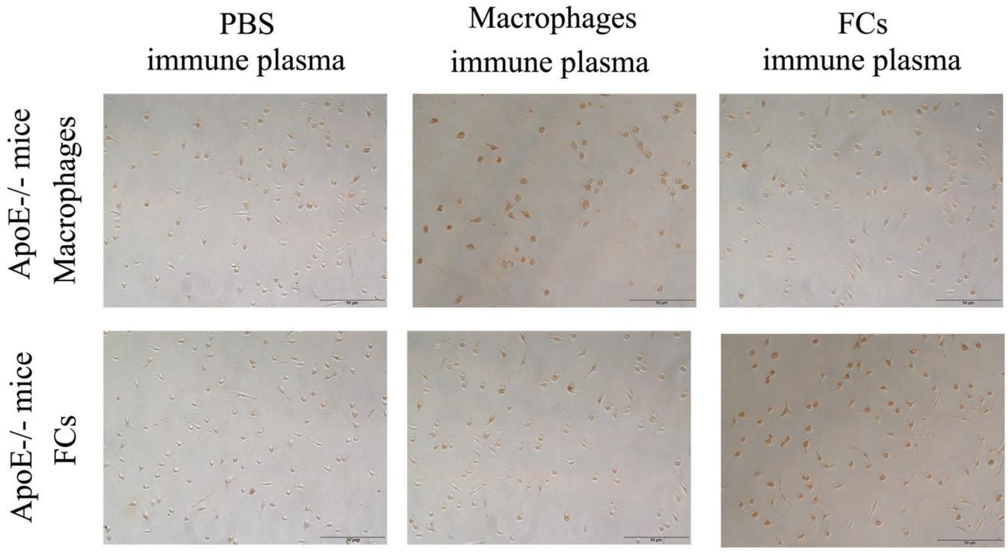

Figure S3 FCs-immune mouse plasma could recognize and bind to FCs from ApoE$^{-/-}$ mice. Peritoneal macrophages and FCs from ApoE$^{-/-}$ mice were fixed and stained with PBS-immune, Macrophages-immune or FCs-immune plasma (dilution 1:50). Scale bars, 50 µm.
